# Potential of *Bacillus pumilus* to directly promote plant growth

**DOI:** 10.3389/fmicb.2022.1069053

**Published:** 2022-12-21

**Authors:** Jakub Dobrzyński, Zuzanna Jakubowska, Barbara Dybek

**Affiliations:** Institute of Technology and Life Sciences—National Research Institute, Falenty, Poland

**Keywords:** spore-forming bacteria, plant growth stimulation, phytohormones, sustainable agriculture, eco-friendly, soil microbiota

## Abstract

Plant Growth-Promoting Bacteria (PGPB) are a promising alternative to conventional fertilization. One of the most interesting PGPB strains, among the spore-forming bacteria of the phylum Firmicutes, is *Bacillus pumilus*. It is a bacterial species that inhabits a wide range of environments and shows resistance to abiotic stresses. So far, several PGPB strains of *B. pumilus* have been described, including *B. pumilus* LZP02, *B. pumilus* JPVS11, *B. pumilus* TUAT-1, *B. pumilus* TRS-3, and *B. pumilus* EU927414. These strains have been shown to produce a wide range of phytohormones and other plant growth-promoting substances. Therefore, they can affect various plant properties, including biometric traits, substance content (amino acids, proteins, fatty acids), and oxidative enzymes. Importantly, based on a study with *B. pumilus* WP8, it can be concluded that this bacterial species stimulates plant growth when the native microbiota of the inoculated soil is altered. However, there is still a lack of research with deeper insights into the structure of the native microbial community (after *B. pumilus* application), which would provide a better understanding of the functioning of this bacterial species in the soil and thus increase its effectiveness in promoting plant growth.

## Introduction

*Bacillus pumilus* is a Gram-positive, spore-forming bacteria, which commonly occurs in various environments including marine water, deep-sea sediments, and soil (Priest, 1993; Shivaji et al., 2006; Liu et al., 2013; Pudova et al., 2022; Yakovleva et al., 2022; Zhang et al., 2022). This species exhibits significant resistance to environmental stresses, e.g., low or no nutrient availability, drought, irradiation, UV radiation, chemical disinfectants, or oxidizing enzymes (Nicholson et al., 2000). Previously, *Bacillus pumilus* was included in the *Bacillus subtilis* group. Currently, *Bacillus pumilu*s belongs to the *Bacillus pumilus* group which also includes *B. altitudinis, B. australimaris, B. safensis, B. xiamenensis*, and *B. zhangzhouensis* (Chen et al., 2016).

Progressive climate change and environmental pollution are intensifying the development of eco-friendly fertilizers (Čimo et al., 2020; Dobrzyński et al., 2021; Kasperska-Wołowicz et al., 2021; Wierzchowski et al., 2021; Zielewicz et al., 2021; Heyi et al., 2022). One of the best solutions for safe fertilization appears to be fertilizers based on plant growth-promoting bacteria (Čimo et al., 2020). Due to its properties, *Bacillus pumilus* is classified as a plant growth-promoting bacteria (PGPB; Gutiérrez-Mañero et al., 2001; De-Bashan et al., 2010; Kaushal et al., 2017). PGPB can stimulate plant growth either directly or indirectly. Mechanisms of direct action are defined as the use of bacterial traits that result in the direct promotion of plant growth, including the production of auxins, e.g., indole-3-acetic acid (IAA), 1-aminocyclopropane-1-carboxylic acid (ACC) deaminase, cytokinins, gibberellins, atmospheric nitrogen fixation (nitrogenase production), phosphorus solubilization, and iron sequestration (by production bacterial siderophores). In contrast, indirect mechanisms relate to the properties of bacteria that inhibit the functioning of one or more plant pathogenic organisms. Indirect mechanisms include, e.g., the production of antibiotics (e.g., cyclic lipopeptides), enzymes that degrade the cell wall of fungi (including chitinases and β-1,3 glucanases), and production of hydrogen cyanide (HCN) inducing plant resistance (e.g., against fungal phytopathogens; Joo et al., 2005; Cuong and Hoa, 2021; Lipková et al., 2021; Shahid et al., 2021; Bessai et al., 2022; Mirskaya et al., 2022)

The mini-review aims to summarize the current state of knowledge on the *Bacillus pumilus* plant growth promotion properties and highlight the lack in the literature on this issue.

### Overall potential of *Bacillus pumilus*

*Bacillus pumilus* is one of the most studied bacterial strains in terms of promoting the growth of bacteria from the genus *Bacillus*. So far, it has been found that *B. pumilus* is capable of producing several phytohormones. Gutiérrez-Mañero et al. (2001) detected a few gibberellins (GA1, GA3, GA4, and GA20) in the culture of *B. pumilus* using full-scan gas chromatography and mass spectrometry assays. Joo et al. (2005) found other gibberellins derived from this species (strain no. CJ-69), including a few new ones such as GA5, GA8, GA34, GA44, and GA5. *B. pumilus* is also able to produce other traits that directly promote plant growth. Isolated from the tea rhizosphere, *B. pumilus* TRS-3 showed the production of indole 3-acetic acid (IAA), siderophore, and phosphate solubilization (Chakraborty et al., 2013). Besides, *B. pumilus* JPVS11 is capable of producing ACC deaminase which contributes to decreasing ethylene levels in the plants by degrading ACC (Kumar et al., 2021). Importantly, *B. pumilus* is also capable of fixing atmospheric N_2_ by nitrogenase production which reduces this nitrogen form to ammonia (Masood et al., 2020). Other authors also detected these plant growth-promoting traits in *B. pumilus* (Hafeez et al., 2006; Murugappan et al., 2013; Upadhyay et al., 2019).

### Promoting plant growth under different growing conditions

To date, several papers have been published on the effect of various *B. pumilus* strains on plant growth parameters. The inoculation efficiency of *B. pumilus* was studied in various conditions including *in vitro*, growth chambers, greenhouses, and in-field conditions. A lot of these studies focus on rice growth promotion and deal mainly with biometric parameters and chemical properties of shoots and roots (Win et al., 2018; Ngo et al., 2019; Liu et al., 2020) Importantly, both commercial strains and soil isolates are used to study on the effects of bacteria on the efficiency in promoting plant growth.

The research based on growing plants on Murashige and Skoog liquid medium has proven that the strain *B. pumilus* LZP02 is able to promote rice growth by increasing the root length, root surface area, number of nodes, root tips, forks, and chlorophyll content (Liu et al., 2020). In addition, the application of *B. pumilus* LZP02 also caused an increase in nitrogen, phosphorus, calcium, and magnesium contents in rice roots (Liu et al., 2020). Previously, it was proven that *B. pumilus* promotes rice growth under growth chamber conditions (Ngo et al., 2019) *B. pumilus* TUAT1 significantly enhanced growth, root development, and nutrient absorption in 21-day-old rice seedlings compared to the control. Interestingly, significantly better efficiency of the studied strain was obtained after inoculating plants with spores than vegetative cells (Ngo et al., 2019).

As well, it has been reported that *B. pumilus* may be a good growth promoter of other plants, including grasses, trees, and others. For instance, after the application of *B. pumilus* of *Alnus glutinosa*, higher values of parameters linked with root system (in both studied soil types) and an increase in shoot surface (in one of the studied soil types) was documented compared to control (Ramos et al., 2003). Subsequently, the application of *B. pumilus* caused an increase in plant height, number of leaves and branches in Chinese tea under *in vivo* conditions (Chakraborty et al., 2013). Moreover, after inoculation of lentils (*Lens culinaris* Medik.) by *B. pumilus*, Siddiqui et al. (2007) noted an increase in plant length and plant fresh weight. Ahmad et al. (2012) also noted that inoculation *B. pumilus* of *Lollium multiflorum* led to increased biomass and growth of plants. Inoculation of wheat (var. Orkhon) by *B. pumilus* led to increasing root length, root area, shoot dry weight, and P and N contents in aboveground plants (Hafeez et al., 2006).

Interestingly, *B. pumilus* can also be an endophytic bacteria. This species was isolated from tissue surfaces of *Ocimum sanctum* and its ability to colonize tissues was confirmed by scanning electron microscopy (SEM; Murugappan et al., 2013). Importantly, the inoculation of *Octimum sanctum* by this species also caused an increase in root and shoot length and leaves number compared to non-inoculated treatment (Murugappan et al., 2013).

Recently it has been suggested that *B. pumilus* promotes plant growth better in combination with nitrogen fertilizers (Win et al., 2018; Masood et al., 2020). Strain *B. pumilus* TUAT-1 with added nitrogen fertilization increased the height, biomass, and chlorophyll content of 21-day-old rice seedlings); (what is important, this study was conducted under field conditions (Win et al., 2018). Next, Masood et al. (2020) carried out a study to determine the main mechanisms relating to PGPB-improved N nutrition in tomatoes under greenhouse conditions. The authors recorded an interesting pattern, namely *B. pumilus* improves tomato growth and N uptake only under N fertilization. Moreover, *B. pumilus* inoculation under nitrogen fertilization increased leaf chlorophyll contents, plant height, shoot fresh weight, and shoot dry weight in comparison with only bacteria inoculation treatment.

It was also documented that *Bacillus pumilus* is able to enhance the activity of a few antioxidant enzymes from the oxidoreductase class, including peroxidase, ascorbate peroxidase, superoxide dismutase, catalase, glutathione reductase, and adenosine triphosphatase in inoculated plants (Liu et al., 2020); (Shahzad et al., 2021). Besides, *B. pumilus* may cause an increase in soil enzyme activity such as alkaline phosphatase, acid phosphatase, urease, and β-glucosidase (Kumar et al., 2021).

### Promoting plant growth under plant stress conditions

*Bacillus pumilus* has also been shown to promote plant growth under abiotic stress conditions (Kumar et al., 2021; Shahzad et al., 2021). Recently, it was found that *B. pumilus* can promote plant growth under salinity stress (Kumar et al., 2021). Positive effects of rice inoculation by *B. pumilus* JPVS11 (pot experiment) such as the enhancement of plant height, root length, and plant fresh weight were observed at the various values of NaCl concentration (0, 50, 100, 200, and 300 mM). Furthermore, *B. pumilus* also may promote plant growth in conditions of Cd contamination; maize seeds inoculation with *B. pumilus* contributed to an increase in the germination percentage, shoot length, leaf length, number of leaves, and plant fresh weight at different concentrations of CdSO_4_ (Shahzad et al., 2021). In addition, Khan et al. (2016) conducted a study on plant growth-promoting properties of rice seedlings by *B. pumilus* under saline and high boron (B) conditions. In non-inoculated treatment, they observed high values of B and salt toxic ions in leaves. On the other hand, there are also studies that show the lack of plant growth promotion by *B. pumilus* under abiotic stress conditions. This phenomenon was found in a study on several plants of the *Brassica genus* under caesium-contaminated conditions (Aung et al., 2015).

### Mechanisms of promoting plant growth

Importantly, inoculation of plants by *B. pumilus* may affect the expression of genes related to root development, for instance, this bacteria increased transcript abundance CRL5 (Murugappan et al., 2013), which regulates crown root formation by expression activation of the OsRR1. It is a gene of rice which encodes a negative regulator of cytokinin signaling (Radhakrishnan et al., 2017). Moreover, *B. pumilus* may decrease transcript abundance WOX11 (Ngo et al., 2019), which contributes to the repression of OsRR2 (Cheng et al., 2016). This fact indicates that the impact of *B. pumilus* on the expression of previously mentioned rice genes may be one of its mechanisms of plant growth promotion (Ngo et al., 2019).

### Plant growth promotion by *Bacillus pumilus* in co-inoculation

There are also results describing the potential to promote plant growth in consortia with other microorganisms. A consortium composed of *B. pumilus* EU927414, *Pseudomonas medicona* EU927412, and *Arthrobacter* sp. EU927410 led to a 24% increase in wheat yield compared to the control under field conditions (Upadhyay et al., 2019). In addition, it has been documented that the application of the consortium of *B. pumilus* and *Bacillus subtilis* increased values of crude protein, dry matter, fat, and carbohydrate in amaranth grains. Besides, in this study, a significant increase in a few amino acid values including methionine lysine and tryptophan was recorded in the studied sample (Pandey et al., 2018). Also dos Santos et al. (2018) used a combined application of *B. pumilus* and *B. subtilis*, however, in sugarcane cultivation. There, together with mineral fertilization and filter cake compost, the solution improved shoot and root growth, as well as increased phosphorus content in soil up to 13% compared to untreated control. Another example of co-inoculation is the application of *B. pumilus* CECT 5105 in combination with *Bacillus licheniformis* CECT 5106 and mycorrhizal fungus *Pisolithus tinctorius* to enhance *Pinus pinea* seedlings growth (Probanza et al., 2001). In this study, authors did not observe a synergic effect with mycorrhizal infection, however, the inoculation by various consortiums showed an increase in a few biometric parameters of the studied plant. For example, *B. pumilus* and *Pisolithus tinctorius* combination led to an increase in aerial and root system parameters.

### Effect of *Bacillus pumilus* on native soil microbiota and post-inoculation tracking of its abundance

A very important aspect related to the application of PGPB is their impact on the indigenous microbiota of the inoculated soil or rhizosphere. Assessing the impact of plant growth-promoting bacteria on the soil microbiota can be crucial to its effectiveness. So far, there are several papers considering the *B. pumilus* effect on the formation of native microbial communities. For instance, (De-Bashan et al., 2010) revealed that inoculation with *B. pumilus* may shift the bacterial community over 60 days under greenhouse conditions and documented that *B. pumilus* prefers to colonize the roots tips and root elongation area (FISH analysis). Besides, using denaturing gradient gel electrophoresis (PCR-DGGE), Kang et al. (2013) conducted a study on the reaction of soil bacterial community soil under fava beans to *B. pumilus* WP8 and its post-inoculation monitoring in soil. Their results indicated that the studied strain survived in large numbers up to 40 days in bulk soil and shifted the bacterial community, especially dominant taxon populations. However, despite the short-lived studied strain in soil, it exhibits the ability to promote fava bean seedlings for at least 90 days; the inoculation of *B. pumilus* WP8 enhanced shoot length, aboveground dry weight, root length, and root dry weight. Interestingly, in the case of another *Bacillus* strain, *Bacillus amyloliquefaciens* NJN-6, using the qPCR technique, (Fu et al., 2017) recorded its stable abundance in the rhizosphere soil of banana plantation within 3 years of inoculation (in the range of 2.5–3.0 log copies of 16S rRNA gene per gram of soil). Also, it is worth mentioning that the survival rate of bacterial inoculants in the soil largely depends on the indigenous soil microbiota; the survival of PGPB in the soil is high when the diversity of native microbiota is low and vice versa (Mallon et al., 2015; Manfredini et al., 2021). In addition, (Bueno et al., 2022) carried out an interesting study on the persistence of *B. subtilis* in the endosphere of soybean roots, showing that after the application of concentrations of 1 × 10^4^ CFU ml^−1^ and 1 × 10^10^ ml^−1^, a higher abundance of this strain was recorded a few weeks after inoculation compared to *B. subtilis* abundance in treatment: *B. subtilis* + mineral fertilization (the study based on transformed *B. subtilis* with ampicillin resistance gene).

The PLFA (phospholipid fatty acid) technique was also used to assess the response of native soil microbiota to *B. pumilus* application. The introduction of a consortium composed of *B. pumilus* and *B. licheniformis* shifted the rhizosphere microbiota, despite the low abundance of both strains in the final phase of the study (Probanza et al., 2001). Another example of using PLFA to evaluate native microbiota reaction to *B. pumilus* inoculation is a study conducted by (Ramos et al., 2003). The authors observed changes in the rhizosphere microbial community in one of the studied soil types from *Alnus glutinosa* cultivation (Ramos et al., 2003).

The only study assessing the status of the native microbiota following the application of *B. pumilus* (strain TUAT-1) was conducted by (Win et al., 2020) using Next-Generation Sequencing (NGS). The researchers studied *B*. *pumilus* TUAT-1 effect on the microbiota of bulk soil, rhizosphere, and root endosphere in forage rice 2 and 5 weeks after transplantation under greenhouse conditions. *B. pumilus* TUAT-1 shifted the microbial community of rhizosphere and roots endosphere, e.g., this bacterial strain significantly contributed to an increase in the Desulfuromonadales abundance and a decrease of the abundance of Xanthomonadales 5 weeks after transplantation of rice. While in the bacterial community of root endosphere, *B. pumilus* TUAT-1 significantly enhanced the relative abundance of Acidobacteriales, Saprospirales, and Alteromonadales 2 weeks after transplanting in comparison with control treatment. However, in bulk soil, the author did not note such a significant alteration in native microbiota after the introduction of the above-mentioned PGPB. Moreover, compared to the control, *B. pumilus* TUAT-1 enhanced bacterial biodiversity, including Shannon diversity in the rhizosphere and root endosphere 2 weeks after transplanting. Importantly, using qPCR techniques, the researchers also demonstrated that *B. pumilus* TUAT-1 persisted in the rhizosphere soil 2 and 5 weeks after transplanting of rice (Win et al., 2020). Different patterns after the introduction of *B. subtilis* into the soil were noted by dos (Dos Santos et al., 2022). The authors showed that *B. subtilis* application did not affect the root endophytic microbial community of soybean (greenhouse conditions), which was also evaluated by alpha diversity metrics. Importantly, it is still unclear whether the PGPB effect on native microbiota can be long-term. This phenomenon may depend on many factors, including the soil’s chemical properties, the stage of plant development, plant root exudates, or indeed the biodiversity and composition of the native microbial community. Hence, there is a need for further studies on the effects of PGPB on indigenous endophytic microbiota and soil microbiota, both for *B. pumilu*s and other strains of the *Bacillu*s genus (Manfredini et al., 2021).

## Conclusion

*Bacillus pumilus* is a very interesting PGPB strain that has already been incorporated into the commercial circuit. Despite that, there are deficiencies in the literature in several areas. There is still a lot to be understood about the reaction of various plants to the inoculation of *B. pumilus*. The need for further research in this field is determined by the specificity of the plant rhizosphere microbiome, which can interact differently with *B. pumilus* strains and vice versa. In addition, it is vital to test the effectiveness of this bacterium on different soil types with different physicochemical properties. Importantly, there is a very small number of studies assessing the impact of *B. pumilus* on the native microbiota using NGS (only one paper to date). NGS offers the possibility of a more detailed analysis of the bacterial community structure than DGGE or PLFA. Hence, it is possible to find the abundance of important taxa involved in biochemical changes in the soil. e.g. whether the abundance of oligotrophic bacteria, including Acidobacteria, decreased after inoculation with the *B. pumilus* strain. Also, in order to test the effect of alterations in the native microbiota under the influence of PGPB, studies of this type should be conducted over a long period of time (even up to several years) and under field conditions. Finally, it is worth mentioning that there is also a lack of studies on the tracking of *B. pumilus* strains in soil (bulk and rhizosphere soil) or plant tissues after its introduction, especially in the long term aspect.

## Author contributions

JD contributed to conception of the minireview. JD, ZJ, and BD wrote the first draft of the manuscript. All authors contributed to manuscript revision, read, and approved the submitted version.

## Conflict of interest

The authors declare that the research was conducted in the absence of any commercial or financial relationships that could be construed as a potential conflict of interest.

## Publisher’s note

All claims expressed in this article are solely those of the authors and do not necessarily represent those of their affiliated organizations, or those of the publisher, the editors and the reviewers. Any product that may be evaluated in this article, or claim that may be made by its manufacturer, is not guaranteed or endorsed by the publisher.
